# Comparison of transfacet and pedicle screws in oblique lateral interbody fusion for single-level degenerative lumbar spine diseases: a retrospective propensity score-matched analysis

**DOI:** 10.1186/s12893-022-01880-w

**Published:** 2022-12-15

**Authors:** Zhao Lang, Tenghui Ge, Jingye Wu, Qiang Yuan, Yuqing Sun

**Affiliations:** Department of Spine Surgery, Peking University 4th Clinical Medical College, Beijing Jishuitan Hospital, No. 31, Xinjiekou East Street, Xicheng District, Beijing, 100035 People’s Republic of China

**Keywords:** Oblique lateral interbody fusion, Disc height, Segmental lordotic angle, Pedicle screw, Transfacet screw, Degenerative lumbar disease

## Abstract

**Background:**

To perform a comparative assessment of percutaneous transfacet screws (TFS) and percutaneous bilateral pedicle screws (BPS) in oblique lateral interbody fusion (OLIF) for the treatment of single-level degenerative lumbar spine diseases in terms of radiological examinations and clinical outcomes.

**Methods:**

Sixty-six patients who received single-level OLIF with percutaneous supplementary fixation assisted by the robot for the treatment of degenerative lumbar spine diseases were selected. There were 16 cases of OLIF with TFS and 50 cases of OLIF with BPS. The propensity score matching method selected 11 patients in each group with matched characteristics to perform a clinical comparison.

**Results:**

The estimated blood loss was 68.2 ± 25.2 ml in the OLIF with TFS group compared to 113.6 ± 39.3 ml in the OLIF with BPS group (*P* < 0.05). The intervertebral disc height raised from 8.6 to 12.9 mm in the TFS group and from 8.9 to 13.9 mm in the BPS group in the immediate postoperative period, and dropped to 10.8 and 12.9 mm at the twelfth month, respectively (*P* < 0.05). The fusion rates were 91% and 100% for TFS and BPS groups (*P* > 0.05). Quantitative assessments of back/leg pain of the two groups reached a healthy level in the late period of the follow-up.

**Conclusion:**

Both TFS and BPS techniques for the OLIF surgery relieve back pain caused by degenerative lumbar spine diseases. The TFS technique exhibits less blood loss compared with the BPS. A moderate cage subsidence is present in TFS but no complication is reported.

## Background

The oblique lateral interbody fusion (OLIF) is a minimally invasive spinal surgery for the treatment of degenerative lumbar spine diseases [1]. This surgical technique is a muscle-sparing method for lumbar interbody fusion with a large geometrically sized fusion cage, which positively contributes to the restoration of lumbar spinal stability [2]. Implantation of a large intervertebral cage allows the height of the interverbal disc to be raised, and thus stretc.hes the bulging disc and folded ligamentum flavum to eventually enlarge the spinal canal and lateral recess [3]. These mechanisms demonstrate how indirect decompression is achieved in OLIF procedure.

OLIF has a range of clinical benefits including less blood loss, relatively low infection rate, high bone grafting rate, and mild postoperative pain due to the small incision. The percutaneous bilateral pedicle screw (BPS) and percutaneous transfacet screw (TFS) fixations are two posterior fixation techniques applied to the OLIF thanks to their biomechanical superiorities on restoring spinal stability and might retard subsidence and restenosis of intervertebral disc [4–6]. A biomechanical study conducted by Reis et al. found that a lateral lumbar interbody fusion by applying BPS sharply decreased the range of motion. The decrease observed was 91% in flexion, 82% in extension and lateral bending, and 74% in axial rotation compared with intact specimens (*P* < 0.05) [7]. Compared with the conventional open approach, the percutaneous pedicle screw fixation technique reaps clinical benefits including less paraspinal muscle damage, less blood loss, and mild pain [8]. TFS fixation in the lumbar spine was described by King and Boucher in the 1940 ~ 1950 s [9, 10]. Chin et al. confirmed the technical reliability of TFS on the restoration of spinal stability [11]. Ferrara’s study concluded that the TFS developed adequate stiffness for the spine to sustain multiple mechanical loadings such as impact and cyclic loading based on an experimental study on cadavers [12]. Moreover, it was reported that the TFS technique reduced the risk of adjacent segment disease in lumbar spine since no injury of adjacent facets is triggered by this screw placement technique [13].

However, no study has been conducted to compare between percutaneous TFS and BPS as supplementary instrumentation for OLIF. Therefore, the aim of this preliminary study is to perform a comparative assessment of two percutaneous screw fixation techniques for single-level degenerative lumbar spine diseases in terms of radiological examinations and clinical outcomes.

## Methods

### Patients and indications

Patient data were collected from our institution between July 2018 and July 2020. The inclusion criteria of patient selection were set as follows: (1) Degenerative lumbar spine disorders; (2) receiving either percutaneous TFS or percutaneous BPS for the single-level OLIF spinal surgery; and (3) data covering at least 12 months follow-up after surgery. The exclusion criteria were defined as follows: (1) Surgical history in lumbar region; (2) lumbar instability resulting from neoplasm, infection or trauma; (3) spondylotic spondylolisthesis; (4) lumbar scoliosis with Cobb angle more than 20° (refer to the minimally invasive spinal deformity surgery (MISDEF2) algorithm); (5) severe osteoporosis (quantitative computed tomography (CT) value < 60 mg/cm^3^); (6) direct posterior decompression including laminotomy and laminectomy; and (7) obvious intraoperative endplate injury detected by early postoperative CT scans. The study was approved by the ethical committee of the authors’ hospital (ethical approval number: 202004-01).

### Surgical approaches

**Fig. 1:**
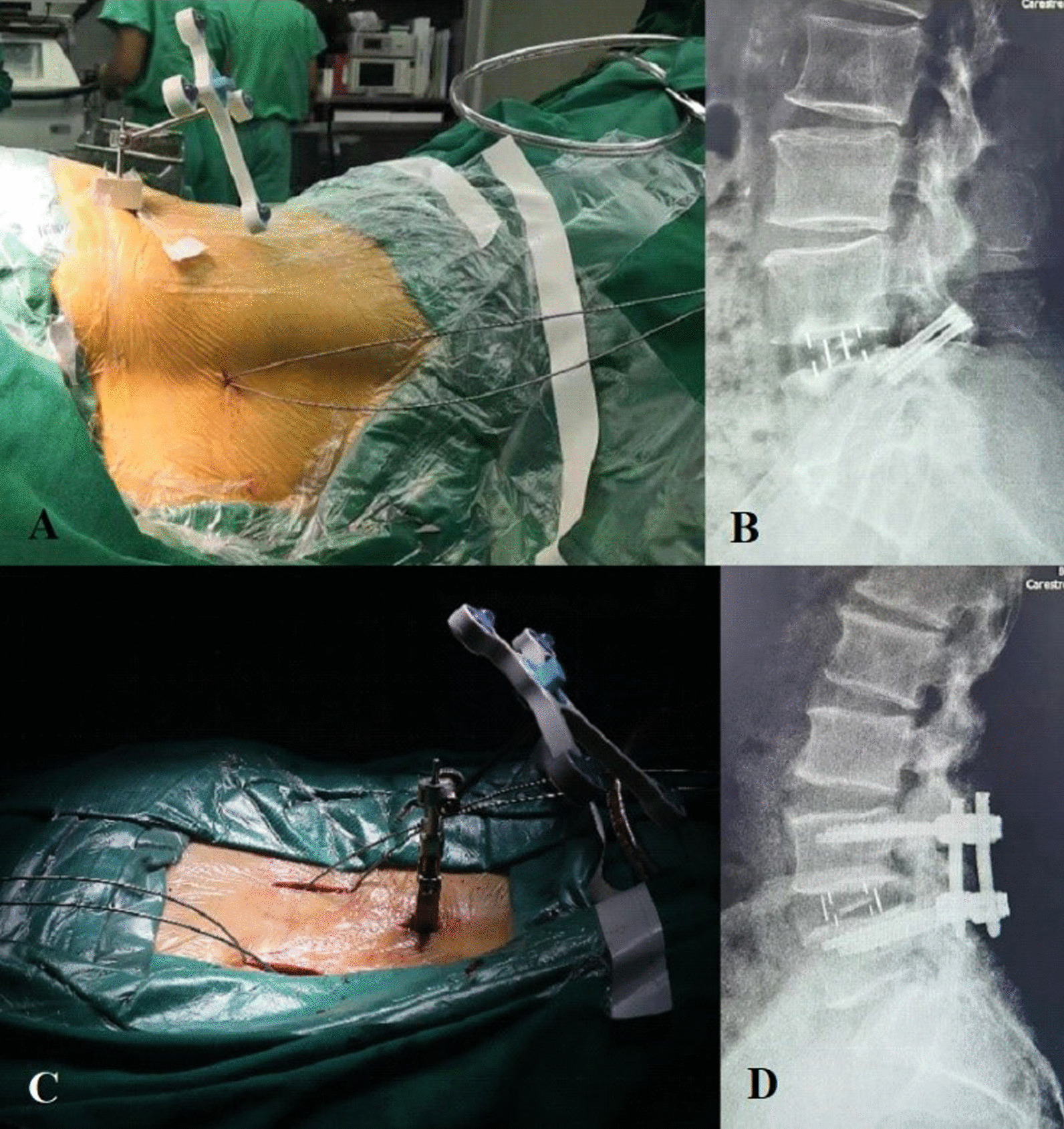
Placement of guiding pins and screws. **A** and **C** Guiding pins were placed percutaneously. The incision for TFS was much less invasive than pedicle screws. **B** and **D** The lateral X-ray of single-level OLIF surgery supplemented by percutaneous TFS or percutaneous BPS at 12-month follow-up

All surgeries were conducted by two experienced spinal surgeons with more than 50 cases of experience in the OLIF surgery. Both of them received the same OLIF surgical training. The OLIF surgery was performed as per the standard protocol with the patient laying down in the right lateral decubitus position. The robot-assisted screw placement technique was used for both the TFS and BPS procedure by employing 3D navigated TiRobot system (TINAVI Medical Technologies, Beijing, China). The robotic arm controlled by a surgeon moved to the target trajectory and laid a guiding pin along the guiding cannula to reach the desired depth. The next step was the insertion of pedicle screws or Herbert screws at the depth measured from the guiding pin (Fig. 1). Since there is no definitive consensus, the choice of supplemental internal fixation for a certain patient was based on the experience and preference of the surgeon. A demineralized allogeneic bone graft (Shanxi OsteoRad Biomaterial Co., Ltd., Shanxi, China) was employed for lumbar intervertebral fusion. Patients were asked to wear a lumbar brace and to do mild out-of-bed activities after surgery. The spinal brace must be worn when patients were out-of-bed for three months after surgery.

### Radiological examination

**Fig. 2:**
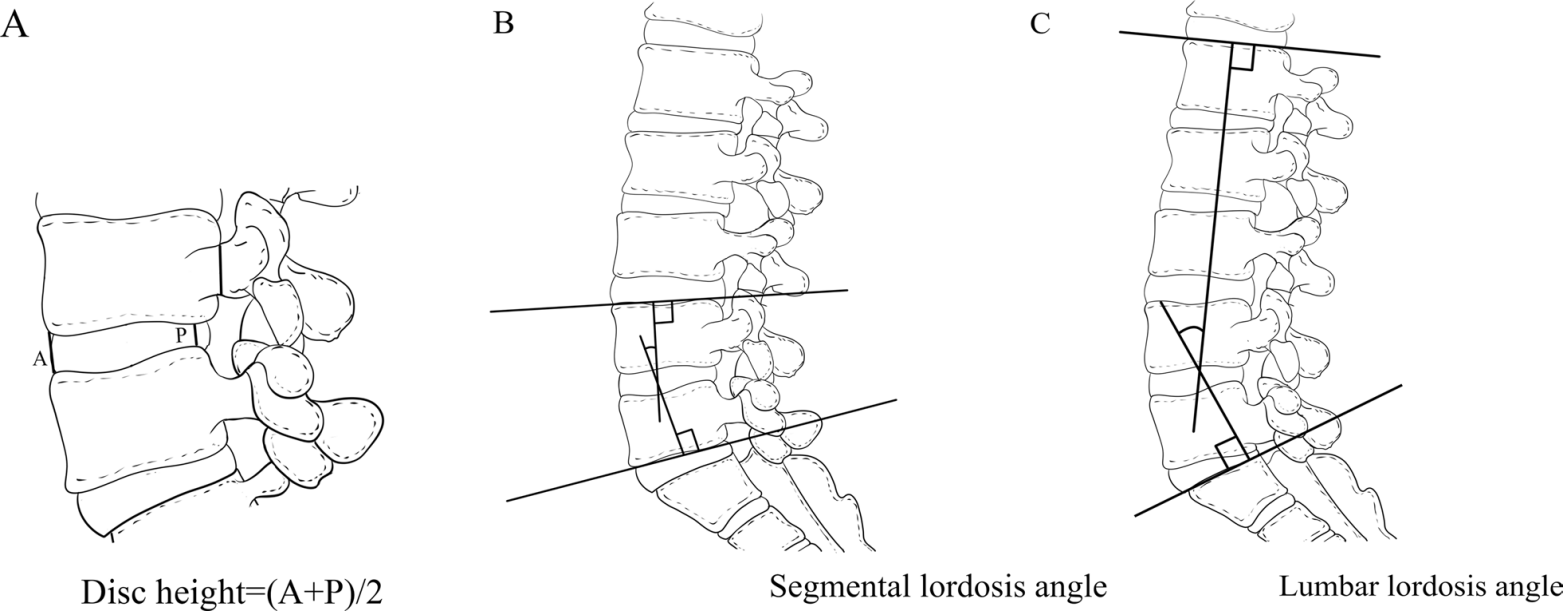
Radiological measurements. **A** The DH was measured on standing neutral lateral radiographs, as an average of the anterior (A) and posterior (P) DHs. **B** The SL angle was defined as the angle between the superior end plate of the upper vertebra and the inferior end plate of the lower vertebra at the corresponding level. **C** The LL angle was defined as the Cobb angle formed between the upper end plate of L1 vertebra and the upper end plate of S1 vertebra

Measurements of geometric features of the lumbar spine were conducted using lateral radiographs of the standing posture and performed at two time points representing two critical stages of rehabilitation of the lumbar spine after surgery: early period after surgery (within 5 days) and twelfth months after surgery. The disc height (DH) was calculated as the mean of the anterior and posterior DHs. The segmental lordotic (SL) angle and lumbar lordotic (LL) angle represented the curvature of a functional spinal unit and the entire lumbar spine. The SL angle was the angle between the superior and inferior endplates of the functional lumbar unit. The measurement angle between the upper endplate of the vertebra L1 and the upper endplate of the vertebra S1 was defined as LL angle (Fig. 2).

Endplate injury and screw accuracy were assessed based on early postoperative CT scans (within 5 days after surgery). Endplate injury was defined as a cage sinking in by > 2 mm from the vertebral endplate using the midline sagittal CT views. Pedicle screw violation was graded as [14]: Grade I, excellent screw position without cortex perforation; Grade II, good screw position with less than 2 mm of cortex perforation. Grade III, poor screw position with more than 2 mm of cortex perforation. The accuracy of transfacet screw was assessed by evaluating the screw entry point with respect to the facet joint and any violation of the pedicle based on Felbaum’s method [15]. They graded the accuracy of an entry point as: transarticular, intraarticular and paraarticular entry. A transarticular entry point was one in which the screw head was on the inferior facet joint. An intraarticular entry point was described as the screw head was more lateral and within the facet joint. And if the screw head was further lateral and purely within the superior facet joint or pedicle of the inferior vertebra, they defined it as paraarticular entry [15]. In terms of pedicle violation of the screw, they graded the accuracy as follows: A, completely within the pedicle; B, pedicle breach within 2 mm; C, pedicle breach between 2 and 4 mm; D, pedicle breach more than 4 mm [15].

The fusion rate was defined as the scale of trabecular bone bridging across the interbody disc based on CT scans 12 months after surgery [16].

### Clinical assessment

Clinical assessment covered operative time, estimated blood loss, hospital stay, obvious complications after surgery and subsequent follow-up. Assessments of management outcomes of lumbar spinal disorder employed three commonly used scoring systems: The Oswestry Disability Index (ODI); Japanese Orthopaedic Association (JOA) scoring system; and Visual Analog Scale (VAS). Two critical time points during follow-up were set for assessment and data collection: third and twelfth months after surgery.

### Statistical analysis

Statistical analysis was conducted by using a commercial software, SPSS 26.0 (SPSS Inc, Chicago, IL). The engagement of propensity score matching method allowed for the creation of matching sets of OLIF surgery with TFS or BPS for selected patients. The multivariable logistic regression model was used for propensity scoring in terms of the following information:

- Patients’ demographic data: age, gender, bone mineral density, body mass index.
- Clinical data: smoking history, interbody fusion cage size (height, length and width), diagnosis, operative level.

The propensity scoring algorithm used one-to-one greedy nearest neighbor matching based on a threshold value of 0.02 to create two groups of patients receiving TFS and BPS of OLIF surgery in each group.

The independent t-test was employed to analyze the data of the two groups without application of propensity score matching. The paired t-test was further used to compare data of two new groups created using the propensity score matching method. The Chi-squared test (*χ*^*2*^-test) and Fisher’s exact test were employed to determine the possibility of nonrandom association between the two groups. The level of statistical significance was set as *P* < 0.05.

## Results

### Demographic and preoperative clinical characteristics

There were 66 patients (25 males and 41 females) selected in this study after inclusion and exclusion criteria were applied. Sixteen patients received TFS and 50 patients received BPS of OLIF surgery. Table 1 shows the baseline characteristics of the patients in each group, where the covariates in each group (operative level of interbody fusion, geometric size of cage, etc.) are significantly different. Performing the propensity score matching procedure, the comparative analysis chose 11 matched pairs of patients in each group. Statistical variables covering bone mineral density, operative level of spinal surgery, geometric size of the cage, reach a matched level via propensity scoring as shown in Table 2. In the two matched groups, all pedicle screws were inserted in prone position, while for transfacet screws, nine cases were performed in lateral decubitus position, the same as OLIF procedure, and the left 2 cases were conducted in prone position.

**Table 1:** Comparison of preoperative clinical characteristics in both groups before propensity score matching

	OLIF with TFS	OLIF with BPS	*P*–value
Patients, No.	16	50	
Age, Mean ± SD, years	62.6 ± 10.2	58.2 ± 11.2	0.163
Sex, No.			0.530
Female	11	30	
Male	5	20	
BMD, Mean ± SD, mg/cm^3^	91.2 ± 21.0	103.1 ± 18.4	0.033*
BMI, Mean ± SD	26.7 ± 3.5	26.2 ± 3.3	0.575
Smoking history, No.			0.625
Yes	5	19	
No	11	31	
Diagnosis, No.			0.578
LS	9	32	
LSCS	7	18	
Level, No.			0.041*
L4/5	13	49	
L3/4	3	1	
Cage, height, No.			0.005*
10 mm	1	6	
11 mm	1	0	
12 mm	5	29	
13 mm	2	1	
14 mm	3	13	
15 mm	4	1	
Cage, length, No.			0.626
45 mm	1	8	
50 mm	8	20	
55 mm	7	22	
Cage, width, No.			0.010*
18 mm	10	46	
22 mm	6	4	

*P-value < 0.05

*OLIF* oblique lateral interbody fusion, *BPS* bilateral pedicle screw; *TFS* transfacet screw, *BMI* body mass index, *BMD* bone mineral density, *LS* lumbar spondylolisthesis, *LSCS* lumbar spinal canal stenosis, *SD* standard deviation

**Table 2:** Comparison of preoperative clinical characteristics in both groups after propensity score matching

	OLIF with TFS	OLIF with BPS	*P*-value
Patients, No.	11	11	
Age, Mean ± SD, years	59.8 ± 11.0	62.6 ± 7.2	0.380
Sex, No.			1.000
Female	8	8	
Male	3	3	
BMD, Mean ± SD, mg/cm^3^	96.7 ± 22.1	95.4 ± 16.7	0.861
BMI, Mean ± SD	25.9 ± 3.0	25.7 ± 3.4	0.881
Smoking history, No.			1.000
Yes	3	3	
No	8	8	
Diagnosis, No.			1.000
LS	6	7	
LSCS	5	4	
Level, No.			1.000
L4/5	10	11	
L3/4	1	0	
Cage, height, No.			0.875
10 mm	1	1	
11 mm	1	0	
12 mm	3	4	
13 mm	1	0	
14 mm	3	5	
15 mm	2	1	
Cage, length, No.			0.670
45 mm	0	2	
50 mm	6	5	
55 mm	5	4	
Cage, width, No.			1.000
18 mm	9	8	
22 mm	2	3	

*P-value < 0.05

*OLIF* oblique lateral interbody fusion, *BPS* bilateral pedicle screw, *TFS* transfacet screw, *BMI* body mass index, *BMD* bone mineral density, *LS* lumbar spondylolisthesis, *LSCS* lumbar spinal canal stenosis, *SD* standard deviation

### Perioperative characteristics

The estimated blood loss was 68.2 ± 25.2 ml in the TFS group and 113.6 ± 39.3 ml in the BPS group (*P* = 0.024). The average operative time for the TFS group was 180.0 ± 47.0 min as compared to 193.6 ± 33.2 min for the BPS group (*P* = 0.414). The average length of hospital stay was 10.0 ± 3.3 days for the TFS group and 11.6 ± 3.8 days for the BPS group (*P* = 0.383) (Table 3).

**Table 3:** Comparison of Perioperative Characteristics and Fusion Rates in both groups

	OLIF with TFS	OLIF with BPS	*P*-value
Patients, No.	11	11	
Operative time, Mean ± SD, min	180.0 ± 47.0	193.6 ± 33.2	0.414
Estimated blood loss, Mean ± SD, ml	68.2 ± 25.2	113.6 ± 39.3	0.024*
Hospital stay, Mean ± SD, days	10.0 ± 3.3	11.6 ± 3.8	0.383
FU duration, Mean ± SD, months	18.1 ± 7.4	18.4 ± 7.2	0.916
Fusion rate at 1y FU, %	90.9	100	1.000

*P-value < 0.05

*OLIF* oblique lateral interbody fusion, *BPS* bilateral pedicle screw, *TFS* transfacet screw, *FU* follow-up, *SD* standard deviation

### Radiological examination

#### DH and lumbar curvature

The mean follow-up duration was 18.2 ± 7.1 (12–31) months. The intervertebral DH raised from 8.6 to 12.9 mm (*P* < 0.001) in the TFS group and from 8.9 to 13.9 mm (*P* < 0.001) in the BPS group in the immediate postoperative period. The DH dropped to 10.8 mm (*P* = 0.002) and 12.9 mm (*P* = 0.028) in the TFS and BPS groups at the twelfth month, respectively. The modifications of lumbar curvature angles of SL and LL in both groups were less than 5% in the immediate postoperative period compared to the preoperative stage, as well as to the twelfth month after surgery (Table 4).

**Table 4:** Comparison of radiographic outcomes in both groups perioperatively and at 1 year follow-up

		OLIF with TFS (n = 11)		OLIF with BPS (n = 11)		*P*-value	
DH (mm)							
Preop		8.6 ± 1.7		8.9 ± 1.9		0.673	
Immediate Postop w/in 5d		12.9 ± 2.4		13.9 ± 2.3		0.321	
*P*-value		< 0.001^†^		< 0.001^†^			
1y Postop		10.8 ± 1.6		12.6 ± 1.8		0.029*	
*P*-value		0.002^‡^		0.028^‡^			
SL (°)							
Preop		16.1 ± 9.1		18.7 ± 4.9		0.453	
Immediate Postop w/in 5d		16.8 ± 8.8		19.3 ± 3.4		0.477	
* P*-value		0.614		0.689			
1y Postop		17.0 ± 8.3		19.6 ± 2.5		0.307	
*P*-value		0.928		0.747			
LL (°)							
Preop		39.3 ± 9.7		39.0 ± 11.7		0.958	
Immediate Postop w/in 5d		40.3 ± 7.6		39.4 ± 12.7		0.851	
* P*-value		0.620		0.772			
1y Postop		44.4 ± 7.6		43.6 ± 9.8		0.858	
* P*-value		0.081		0.053			

^†^*P*-value < 0.05 preoperative vs. immediate postoperative within 5 days

^‡^*P*-value < 0.05 immediate postoperative within 5 days vs. 1 year postoperative

*P-value < 0.05 OLIF with TFS vs. OLIF with BPS

*OLIF* oblique lateral interbody fusion, *BPS* bilateral pedicle screw, *TFS* transfacet screw, *DH* disc height, *SL* segmental lordosis, *LL* lumbar lordosis

#### Assessment of screw accuracy

Over 40% (41, 93%) of screws in the BPS group were scored as grade I, followed by grade II (3, 7%). In the TFS group, all screws had a transarticular entry point. As to violation of the pedicle, eighteen out of 22 (82%) screws, were identified as grade A of accuracy. The rest four screws (18%) penetrated the cortex within < 2 mm and scored as grade B of accuracy. No screw-induced complications were reported.

### Clinical outcomes

At the twelfth month, the fusion rates were 91% (10/11) and 100% (11/11) for groups TFS and BPS, respectively. Quantitative assessments of back/leg pain, ODI and JOA exhibit sharp variations between the preoperative stage and the twelfth month after surgery. The value of ODI improved by 81% in the TFS group and 79% in the BPS group. The value of the JOA increased by 57% in the TFS group, and 82% in the BPS group. The value of the VAS for the evaluation of back pain fell more than 85% both in the TFS group and BPS group. In terms of the assessment of leg pain based on a VAS, the value dropped around 90% both in the TFS group and BPS group (P < 0.05) (Table 5).

**Table 5:** Comparison of clinical outcomes in both groups

	OLIF with TFS (n = 11)	OLIF with BPS (n = 11)	*P*-value
ODI			
Preop	47.0 ± 22.8	50.6 ± 15.5	0.669
3m Postop	24.1 ± 19.2	29.0 ± 14.4	0.595
*P*-value	0.002†	0.001†	
1y Postop	8.8 ± 7.8	10.4 ± 10.7	0.611
*P*-value	<0.001^‡^	<0.001^‡^	
JOA			
Preop	16.8 ± 3.9	14.0 ± 2.2	0.101
3m Postop	23.1 ± 4.6	20.1 ± 5.1	0.234
*P*-value	0.001^†^	0.001^†^	
1y Postop	26.3 ± 2.6	25.5 ± 2.8	0.192
*P*-value	<0.001^‡^	<0.001^‡^	
VAS (back)			
Preop	5.3 ± 2.4	5.6 ± 2.9	0.819
3m Postop	1.3 ± 2.0	1.4 ± 1.5	0.882
*P*-value	<0.001^†^	<0.001^†^	
1y Postop	0.6 ± 0.8	0.4±0.8	0.391
*P*-Value	<0.001^‡^	<0.001^‡^	
VAS (leg)			
Preop	3.6 ± 2.5	5.1 ± 1.9	0.075
3m Postop	1.5 ± 2.2	1.0 ± 1.8	0.638
*P*-value	0.041^†^	<0.001^†^	
1y Postop	0.3 ± 0.6	0.4 ± 0.5	0.756
*P*-Value	0.001^‡^	<0.001^‡^	

^†^*P*-value < 0.05 preoperative vs. 3 months postoperative

^‡^*P*-value < 0.05 preoperative vs. 1 year postoperative

*OLIF* oblique lateral interbody fusion, *BPS* bilateral pedicle screw, *TFS* transfacet screw, *ODI* Oswestry Disability Index, *JOA* Japanese Orthopaedic Association, *VAS* visual analog scale

One patient complained of transient sympathetic dysfunction symptoms (no sweat in left lower extremity) in the TFS group, but the symptom was relieved during the follow-up period.

## Discussion

The percutaneous TFS placement applied to the OLIF surgery gained clinical benefit with significantly less intraoperative blood loss compared with another minimally invasive technique, BPS. Blood loss from the TFS procedure was around 60% of that of the BPS (68.2 ml vs. 113.6 ml), suggesting less damage to the surrounding tissues of the lumbar spine. This finding is consistent with published studies [13]. Previous published studies have demonstrated that the estimated blood loss ranged from 40 to 262.5 ml in surgeries which applied percutaneous pedicle screw fixation, compared to 145–870 ml in open surgeries [17].

Both TFS and BPS techniques had an equivalent operative time and hospital stay. Previous studies reported that the average surgical time of percutaneous TFS and BPS techniques as supplements to OLIF or lateral lumbar interbody fusion was 142 min and 121 min, respectively [18, 19]. The average operative time in the TFS group and BPS group in this study were 180 and 194 min, respectively, slightly longer than the published data. Given that stand-alone OLIF took 104 min on average according to the literature [20], the operative time of OLIF combined with open TFS was about 166–184 min [21, 22], and 164-361 min in OLIF combined with open BPS [17]. Thus, percutaneous surgery did not prolong the overall operative time compared with an open approach. Repositioning, reprepping and redraping were considered by some surgeons as a time-consuming process. However, according to our experience, it only took about 20 min if the operation room staff was skilled. Therefore, we considered the cases with two different intraoperative positions in TFS group as a whole for statistical analysis.

Another clinical superiority for both techniques was the accuracy of screw placement thanks to the engagement of robot-assisted techniques. Our previous studies have demonstrated that the accuracy of the screw position of robot-assisted TFS placement exceeded 90% [23]. Complications caused by screw misplacement (including neurological sequelae, infection, etc.) have been reported in published studies [24]. In the present study, the accuracy of screw placement achieved or exceeded the clinical favorable level, where the cortex perforation was less than 2 mm. Importantly, no complications resulting from screw misplacement were found in all patients during entire follow-up period.

DH was restored in the early postoperative period with either the TFS or BPS technique. The increase rate for both groups was not less than 50%, increasing 4.3 and 5 mm for TFS and BPS, respectively. These results are consistent with previous studies [25, 26]. However, neither group kept favorable clinical outcomes during the entirety of the follow-up period. Moderate settlement of DH was found in both groups at the twelfth month of the follow-up. The DH of the TFS group dropped from 12.9 mm to 10.8 mm (16%), and from 13.9 mm to 12.6 mm (10%) in the BPS group. That said, the increased rate of DH in the TFS and BPS groups are around 26% and 42% with respect to their preoperative data, respectively. As an important assessment item of clinical efficacy for interbody fusion, these two supplementary fixation techniques might lose credits. The BPS technique has a relatively favorable performance on the DH restoration when compared to the TFS counterpart. The percutaneous BPS placement exhibits higher biomechanical strength to sustain the compression exerted by body weight and daily activities compared with TFS placement. Notably, the fusion rate of the two groups was more than 90% at the twelfth month, indicating that it was not affected by the loss of DH in the late period of the follow-up, suggesting a successful bone graft. However, severe cage subsidence (more than 50% collapse into vertebral endplate) can trigger persistent back pain or radiculopathy, as a result, a revised spinal surgery is required [27]. Tempel et al. also reported the data of subsidence grade for a revised spine surgery. They defined subsidence grading as the percentage of disc space or vertebral body collapse around the interbody graft compared with the immediate postoperative films: Grade 0, 0–24% collapse; Grade 1, 25–49% collapse; Grade 2, 50–74% collapse; and Grade 3, 75-100% collapse. They found the median subsidence grade for patients requiring revision surgery was 2.5 [28]. Fortunately, in our study, the amount of subsidence in the TFS group (16%) was at a low level, and the likelihood of occurrence of related complications would be low. The assessment in terms of multiple evaluation systems (VAS, JOA, and ODI) demonstrated favorable clinical efficacy of the two screw placement techniques (Table 5), and further demonstrated the minor adverse effects of moderate cage subsidence as shown in the TFS and BPS groups.

The corrections of curvature at the functional spinal unit level by using two surgical protocols of screw placement (TFS and BPS) were similar and minor (around 1 degree of SL, Table 4). Takayoshi et al. reported on the correction of segmental lordosis with 1.8 degrees, and the management method also employed the OLIF with supplemental percutaneous pedicle screws without posterior decompression [29]. Some published studies found that the OLIF technique was able to restore lumbar segmental lordosis from 1.2° to 3.6° without osteotomy [30]. In contrast, a relatively low level of modification of the lumbar segmental lordosis, around 1°, was found in this study compared with previously published studies. The clinical benefit of correction of segmental lordosis may not be confirmed in this study.

Although weak difference was demonstrated between two techniques in the present study, TFS technique had some unique advantages in clinical practice. Compared with percutaneous BPS technique, the significant reduction in soft tissue damage is an advantage of percutaneous TFS technique, leading to less functional loss of muscle along with decreased infection rate [24]. In addition, the most cephalad screw or rod in the construct of percutaneous pedicle screw fixation may mechanically compromise the adjacent facet [31], which will lead to an increase in the degeneration of adjacent segments [32]. Differently, no injury of adjacent facets is triggered by transfacet screw fixation due to its entry point and direction and unnecessary rod usage [33]. Lastly, Facet fixation is an alternative when fractured or small pedicles are encountered. Of course, more obvious subsidence following percutaneous TFS technique is still a concern, which requires more samples and longer follow-up observations.

A limitation of this single-center study was the small sample size as this may have led to underlying bias. The propensity score matching method might have a positive role to minimize the influence of confounding factors, which made the comparison between the two groups more convincing because of comparable baseline. A prospective study with a large sample size is mandatory in the future to further clarify the optimal choice of supplemental internal fixation after OLIF procedure. In addition, a potential intervention factor existing in the analysis was different clinical outcomes conducted by different surgeons, even though all surgeries were performed by two experienced surgeons.

## Conclusion

Both TFS and BPS techniques for the OLIF surgery achieve successful interbody fusion and relieve back and leg pain caused by degenerative lumbar spine diseases. The TFS technique exhibits a clinical benefit of less blood loss compared with bilateral pedicle screw fixation. A moderate settlement of DH caused by cage subsidence is present in the TFS procedure one year after surgery, but no complication regarding back pain resulting from the cage subsidence is detected in either the TFS or BPS technique.

## Data Availability

The datasets generated and analyzed during the current study are not publicly available due to limitations of ethical approval involving the patient data and anonymity but available from the corresponding author on reasonable request.

## Abbreviations

BMD: Bone mineral density
BMI: Body mass index
QCT: Quantitative computed tomography
OLIF: Oblique lateral interbody fusion
ODI: Oswestry Disability Index
VAS: Visual Analog Scale
JOA: Japanese Orthopedic Association
DH: Disk height
SL: Segmental lordosis
LL: Lumbar lordosis
BPS: Bilateral pedicle screw
TFS: Transfacet screw
